# Uniaxial Negative Thermal Expansion and Mechanical Properties of a Zinc-Formate Framework

**DOI:** 10.3390/ma10020151

**Published:** 2017-02-10

**Authors:** Hongqiang Gao, Wenjuan Wei, Yizhang Li, Rong Wu, Guoqiang Feng, Wei Li

**Affiliations:** 1 School of Physical Science and Technology, Xinjiang University, Urumqi 830046, China; ghqe1@outlook.com; 2 School of Physics, Huazhong University of Science and Technology, Wuhan 430074, China; wwenjuan@hust.edu.cn (W.W.); wl276@hust.edu.cn (W.L.); 3 Department of Civil Engineering, The University of Sheffield, Sheffield S10 2TN, UK; yli169@sheffield.ac.uk; 4 Department of Physics and Mechanical & Electrical Engineering, Hubei University of Education, Wuhan 430205, China

**Keywords:** metal-formate framework, negative thermal expansion, mechanical property

## Abstract

The thermal expansion behavior of a metal-formate framework, Zn(HCOO)_2_·2(H_2_O) (**1**), has been systematically studied via variable temperature single-crystal X-ray diffraction. Our results demonstrate that this formate exhibits significant negative thermal expansion (NTE, −26(2) MK^−1^) along its *c*-axis. Detailed structural analyses reveal that the large NTE response is attributed to the ‘hinge-strut’ like framework motion. In addition, the fundamental mechanical properties of framework **1** have been explored via nanoindentation experiments. The measured elastic modulus and hardness properties on the (00-2)/(100)/(110) facets are 35.5/35.0/27.1 and 2.04/1.83/0.47 GPa, respectively. The stiffness and hardness anisotropy can be correlated well with the underlying framework structure, like its thermoelastic behavior.

## 1. Introduction

Metal-formate frameworks (MFFs) have attracted considerable attention in the past decade due to their rich physical properties which include magnetism [1,2,3], ferroelectricity [3,4,5,6], negative linear compressibility [7], mechanical properties [8,9,10,11], and dielectricity [12,13]. Very recently, a few research groups have discovered that these MFFs can also exhibit large negative thermal expansion (NTE) properties [14,15,16,17,18]. For instance, Shang et al. reported a niccolite-like MFF, [NH_3_(CH_2_)_4_NH_3_][Mg_2_(HCOO)_6_] in 2013, which exhibits colossal NTE of about −648 MK^−1^ along its *b*-axis when it crosses a monoclinic to trigonal phase transition [17]. Such giant NTE response arises from the cooperative coupling of the librational motions of the organic amine cations and the magnesium-formate framework modulation, which is in marked contrast to the well-known transverse vibration mechanism responsible for NTE in traditional materials (i.e., ZrW_2_O_8_) [19,20,21]. In 2015, Collings et al. studied the thermal expansion behavior of a series of MFFS with ABX_3_ perovskite architecture and discovered the compositional dependence of anomalous thermal expansion in these formats [14]. Notably, the size of A-site cations and their hydrogen bonding modes with the host frameworks, have been shown to play a very crucial role in determining the NTE magnitudes in these formate perovskites. The strongly hydrogen-bonded [(NH_2_)_3_C][M(HCOO)_3_] family gives almost an order of magnitude lower NTE response compared with the weakly hydrogen bonded system [CH_3_NH_3_][M(HCOO)_3_] (M = Mg^2+^, Mn^2+^, Co^2+^, Ni^2+^, Cu^2+^, Zn^2+^ and Cd^2+^) [22]. These examples have clearly shown the great potential of MFFs as new NTE materials. Nevertheless, most reported NTE MFFs are cation-anionic type frameworks ([AmineH^n+^][M(HCOO)_3_]_n_) in which the NTE arises from the cooperative motion of both organic amine cations and anionic framework. To fully explore this exciting field, the NTE behavior of MFFs with neutral framework architecture also needs to be considered. Herein, we present the thermal expansion study of a MFF, Zn(HCOO)_2_·2(H_2_O) (**1**), and elucidate its NTE mechanism from a molecular level. In addition, we report the elastic and hardness properties of this MFF and explain the mechanical anisotropy from a viewpoint of framework structure.

## 2. Results and Discussion

### 2.1. Crystal Structure Description

**Figure 1:**
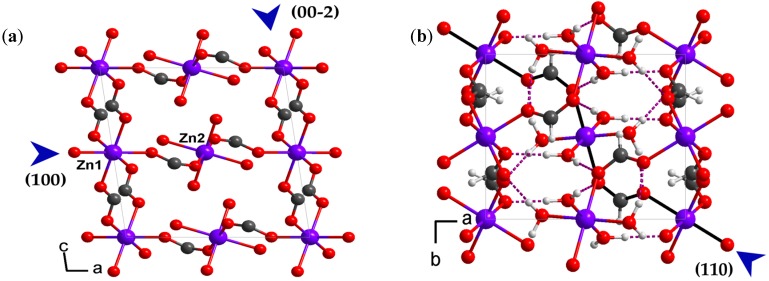
Framework structure of **1** viewed normal to the (010) (**a**) and (001) planes (**b**). Hydrogen atoms were omitted in (**a**) and the black linkages along the <110> direction in (**b**). Color scheme: Zn, purple; O, red; C, dark grey; H, 25% grey. Blue arrows represent the indentation directions.

Framework **1** crystallizes under ambient conditions in the *P*2_1_/*c* monoclinic space group and has a 3-D network constructed by formates, as previously reported [23]. In the structure, there are two crystallographically independent metal centers coordinated by formate ligands and water molecules [24,25]. Both the zinc ions show a slightly distorted octahedral coordination: Zn1 is coordinated by six formate ligands, and Zn2 is surrounded by two formates and four water molecules (Figure 1a) [23]. These two types of zinc octahedral are linked by bitopic formates and waters to form a 3-D framework. There are two crystallographically independent coordination waters, and they form four independent hydrogen bonds with formate ligands of the framework (Figure 1b).

### 2.2. Thermal Expansion Study

**Figure 2:**
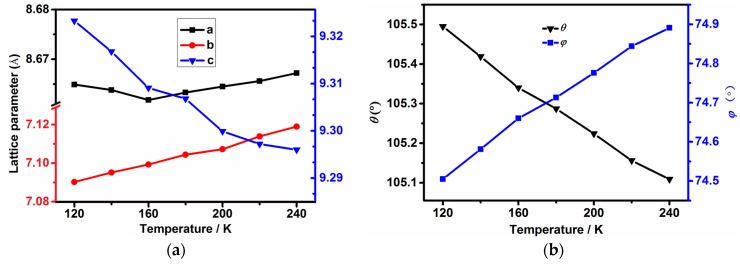
(**a**) Temperature-dependent principle axis parameters X_1_, X_2_ and as a function of temperature; and (**b**) The temperature dependent variations of hinge angle θ and ϕ.

The thermal expansion behavior of framework **1** is studied via variable-temperature single crystal X-ray diffraction experiments. The obtained lattice parameters were then used as input for *PASCal* program in order to determine the thermal expansion coefficients of framework **1**, and the thermal expansivity indicatrix of **1** is given in Figure S3 [26]. As shown in Table S1, X_1_ and X_2_ axes are approximately along the *c*- and *a*-axis, and X_3_ is along the *b*-axis. The X_3_ principle axis length undergoes the most significant change upon temperature perturbation, which exhibits positive thermal expansion (PTE) by about 0.4% increase from 120 to 240 K (Figure 2a). In contrast, the X_1_ axis length shows negative thermal expansion (NTE) upon heating and decreases by −0.3% in the measured temperature range. Interestingly, the X_2_ principle parameter only exhibits trivial change of about 0.06% in the measured temperature range. And the relevant variations of the volume and beta angle are about 0.1% and −0.09%. The average coefficients of thermal expansion along the X_1_, X_2_ and X_3_ principle axes are 5.3(1.5), 33(1), and −26(2) MK^−1^, respectively; and the volumetric thermal expansion coefficient is 21(3.7) MK^−1^. The NTE coefficient of framework **1** has the similar magnitude of those from the perovskite-like MFFs, [CH_3_NH_3_][M(HCOO)_3_] (M = Mg, Mn, Fe, Co, Zn and Cd), [(CH_3_)_2_NH_3_][Cu(HCOO)_3_], [C(NH_2_)_3_][Cd(HCOO)_3_], but about five times higher than those from [C(NH_2_)_3_][M(HCOO)_3_] (M = Mn, Fe, Co and Zn) and [NH_2_NH_3_][Er(HCOO)_4_] as seen from Table 1 [14,18]. In comparison with the NTE coefficients across phase transitions of niccolite-like [NH_3_(CH_2_)_4_NH_3_][Mg(HCOO)_6_] (α*_c_* ≈ −170 MK^−1^, α*_b_* ≈ −648 MK^−1^) [17], perovskite-like [NH_2_NH_3_][M(HCOO)_3_] (M = Mn, α*_a_* ≈ −96 MK^−1^; M = Zn, α*_a_* ≈ −108 MK^−1^) and [NH_2_NH_3_][M(HCOO)_3_] (M = Co, α*_b_* ≈ −81 to −100 MK^−1^; M = Mg, α*_b_* ≈ −69 to −89 MK^−1^) with 4^9^·6^6^ framework topology [19], the NTE response of **1** is significantly smaller since the drastic structural changes across phase transitions play a pivotal role in determining thermal expansion in these MFFs.

**Figure 3:**
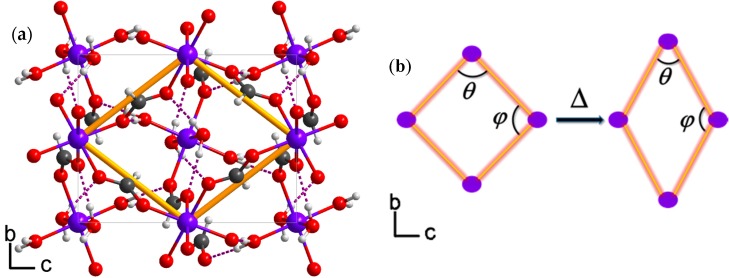
Thermal expansion mechanism of framework **1**. (**a**) The yellow framework frames represent ‘hinge-strut’ like structure and violet dotted lines represent hydrogen bonds. Color scheme: Zn, purple; O, red; C, dark grey; H, 25% grey; and (**b**) The decreasing of hinge angle θ, from 105.5° at 120 K to 105.1° at 240 K, and coupled with the ϕ increased from 74.5° at 120 K to 74.9° at 240 K upon heating.

To understand the NTE mechanism and elucidate the underlying structural origin, the collected single-crystal X-ray diffraction data were solved and the atomic coordinates at each temperature were carefully compared. In order to understand the NTE behaviour of **1**, we simplified the framework as a ‘hinge-strut’-like architecture which is a prototypical structural motif responsible for anisotropic thermal expansion in framework materials [27]. In this simplified model, the formate ligand and Zn^2+^ ions represent the hinge and strut, and the hinge angles are denoted as θ and ϕ [18,28]. Upon heating from 120 to 240 K, the Zn-O bond lengths and O-Zn-O angles only show trivial changes (Table 2), and three independent hydrogen bond lengths do not exhibit obvious alterations either. However, the O5-H4⋯O3 bond (shown in Figure S4) expands from 2.726(3) to 2.746(3) Å and all the O-H⋯O angles decrease significantly upon heating as seen in Table 2. Such cooperative structural variations result in the distortion of the zinc octahedra and twist of the formate ligands. Though the distortion and twist of each octahedral and formate linker are generally small, the accumulated effect in the ‘hinge-strut’ model become significant, hence inducing the decrease of θ but increase of ϕ from 120 to 240 K. As shown in Figure 3b, θ decreases from 105.5(0)° at 120 K to 105.11(0)° at 240 K, and ϕ increases from 74.51(0)° to 74.89(0)° upon heating in the same temperature range. Such alterations give rise to NTE along the X_1_ axis coupled to the large PTE along the X_3_ axis. However, the changes of hydrogen-bonding do not show any obvious impact perpendicular to the ‘hinge-strut’ structure, thus leading to small thermal expansion response along the X_2_ axis. Finally, we can uncover the reason why the NTE magnitude of **1** is similar to many MFFs which have all their MO_6_ octahedra fully linked by formate ligands. Though framework **1** contains half of its zinc octahedra linked by only two formates and four water molecules, the abundant hydrogen-bonding strengthens the framework’s resistance to variations in temperature.

### 2.3. Mechanical Properties

**Figure 4:**
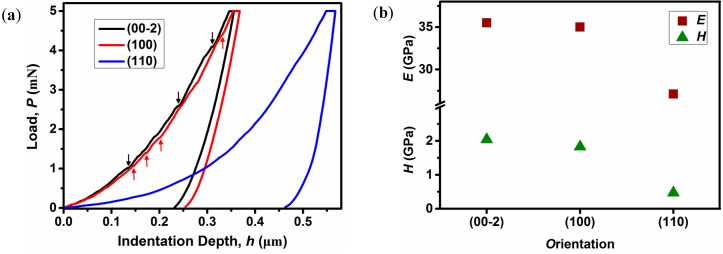
(**a**) Representative *P*-*h* curves of framework **1** measured along the three different directions with a maximum indentation load of 5 mN using a Berkovich tip. Note that the arrows represent the ‘pop-ins’ or displacement bursts from the (00-2) and (100) planes; and (**b**) The calculated elastic modulus and hardness properties derived from the *P*-*h* curves in dependence of orientation.

Nanoindentation experiments were conducted using a Berkovich tip with a radius of ~50 nm in quasi-static mode [29,30,31], and representative load-indentation depth (*P*-*h*) curves obtained on the three different natural facets, (00-2), (100), and (110), are shown in Figure 4a. The load segments of the *P*-*h* curves on the (00-2) and (100) facets show some small discontinuities (‘pop-ins’), indicating that the plastic deformation which occurs underneath the indenter tip, is heterogeneous in nature. In contrast, the indentation induced plasticity is relatively homogeneous on the (110) face [32].

The elastic modulus (*E*) and hardness (*H*) properties were calculated using the Oliver-Pharr method and the average values are shown in Figure 4b [33,34]. The *E* and *H* values of (110), (100), and (00-2) faces are 27.1(5), 35.0(9), 35.5(6) GPa, and 0.47(3), 1.83(8), 2.04(8) GPa, respectively. The modulus shows medium anisotropy with *E*_(110)_/*E*_(100)_/*E*_(00-2)_ = 1/1.292/1.310, while the hardness exhibits much larger anisotropy with *H*_(110)_*/H*_(100)_*/H*_(00-2)_ = 1/3.894/4.340. The anisotropic mechanical properties of **1** can be rationalized by referring to its underlying framework structure. In the structure, each Zn1O_6_ octahedron is connected to the next Zn1O_6_ octahedron via two formate ligands along the *c*-axis, while each Zn1O_6_ octahedron is linked to the neighboring Zn2O_6_ octahedron alternatively only via a single formate along the *a*-axis. As expected, the former dense connection mode exhibits higher resistance against elastic and plastic deformation during indentation, hence, giving rise to slightly higher elastic modulus and hardness. In contrast, the later loose linkage mode tends to be more easily deformed by the berkovich indenter tip, thus showing lower mechanical strength. When indenting normal to the (110) plane (Figure 1b), the indenter tip is not along the direction of the strong formate linkages, instead of a 45° angle, much lower stiffness and hardness properties were observed.

As listed in Table 3, the average *E* and *H* values of framework **1** are larger than most known MFFs in general. The moduli of **1** are only slightly higher than those from [NH_4_][Zn(HCOO)_3_], while its stiffness anisotropy is significant lower. In comparison with the ABX_3_ perovskite-like MFFs which have similar framework density and strong hydrogen-bonding between the amine cations and formate frameworks, such as [NH_3_NH_2_][Zn(HCOO)_3_] and [C(NH_2_)_3_][Mn(HCOO)_3_], the *E* and *H* values of framework **1** are about 15%–24% and 50%–65% higher depending on different crystallographic orientations (by taking no account into the (110) face). However, the *E* and *H* values of framework **1** are about two to three times of those from the less hydrogen-bonded formate perovskite-like MFF, [(CH_2_)_3_NH_2_][Mn(HCOO)_3_] [22], which although have large hydrogen-bond strength but duo to the presence of only one NH_2_ for this compound compared with guanidinium analogue. Though the rare earth MFF, [NH_2_CHNH_2_][Er(HCOO)_4_], exhibits about 17% higher density, its highest *E* and *H* values are about 18% and 11% lower [18]. The reason could be attributed to the relatively weaker Er-O bonding in [NH_2_CHNH_2_][Er(HCOO)_4_] compared to Zn-O bonding in framework **1**.

**Figure 5:**
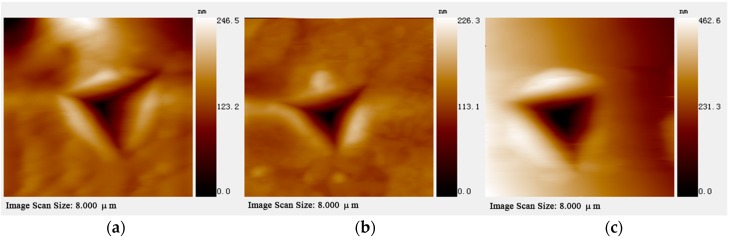
The atomic force microscope images of the residual indents obtained from the indenter normal to the (00-2) (**a**); (100) (**b**); and (110) (**c**) planes of framework **1**.

**Figure 6:**
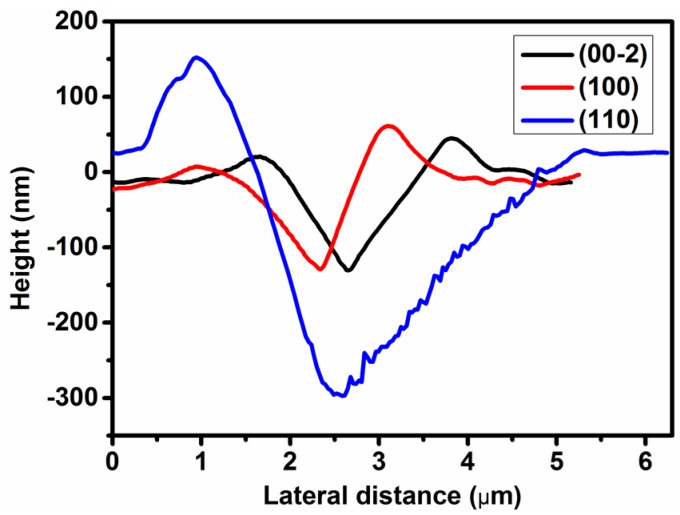
The corresponding cross-sections at the center of the residual indent impressions on (00-2), (100), and (110) facets.

The atomic force microscopy (AFM) images of the residual impressions upon unloading on the three faces are shown in Figure 5. It can be seen that the indent impression on all faces shows ‘pile-up’ signature, which arises from incompressible plastic deformation of material from beneath the indenter to the top surface at the periphery [32]. Moreover, the quantity and shape of ‘pile-ups’ produced along the edges of the indenter tip are strongly dependent on the crystallographic orientation. As shown in Figure 6, the (00-2) and (100) planes exhibit ‘pile-ups’ with height of about 50 and 40 nm. In contrast, the height of ‘pile-ups’ in the (110) plane is about 150 nm, which is about 3–4 times of those from the other two faces. As mentioned above, the significantly weak linkage along the (110) plane gives rise to its low hardness, hence, large amounts of plastically-deformed framework components would appear during indentation. Since the less connected Zn2O_6_ octahedra could be ruptured more easily by the indentation stress compared with the strongly linked Zn1O_6_ octahedra, they could dislocate and displace more readily around the indentation periphery, hence, resulting more significant ‘pile-ups’ along (110) plane. 

## 3. Materials and Methods

### 3.1. Synthesis

All reagents were commercially available and used as received. The compound was prepared using the conventional hydrothermal methods. The typical process for synthesis: 0.3038 g zinc nitrate hexahydrate (1 mmol) was dissolved in the mixed solution of 4 mL water and 6 mL *N*,*N*-dimethylformamide (DMF) and transferred to a 15 mL Teflon-lined autoclave. The mixture was heated to 80 °C for 72 h in an oven. Colourless block-shaped crystals were filtered from the mother liquor, washed with DMF (3 mL × 2) and dried in air. The purity of the samples was confirmed by the powder X-ray diffraction (PXRD) spectra (Figure S1).

### 3.2. Variable-Temperature Single Crystal X-ray Diffraction

Single-crystal X-ray diffraction experiments were performed at a nitrogen stream condition using an Oxford Diffraction Gemini E Ultra diffractometer (Rigaku Oxford Diffraction Ltd., Oxofrd, UK) with an Eos CCD detector. The crystals were glued on a glass fiber for measurement. Data were collected using Mo-K*α* radiation (λ = 0.71073 Å) by the ω scan approach at a temperature between 120 and 240 K at intervals of 20 K. Data collection, cell determination and refinement and data reduction were applied with *CrysAlis^Pro^* program. The structures were solved and refined using the direct method and full matrix least-squares procedure on *F*^2^ by the SHELXS and SHELXL 97 programs [35]. All non-hydrogen atoms with anisotropic thermal parameters were refined and all hydrogen atoms were located from the electron density map and refined using a riding mode and isotropic displacement parameters constrained to 1.2 times those of their adjacent carbon or oxygen atoms.

### 3.3. Nanoindentation Experiment

Nanoindentation experiments on the single crystal of framework **1** were performed at room temperature using a Hysitron TI 750 Ubi Triboindenter (Hysitron Corp., Minneapolis, MN, USA), equipped with an *X*/*Y* and *Z*-axis staging system. The instrument was placed in an acoustic enclosure for minimizing the interference of acoustic noise, block air currents and shielding against thermal instability. The transducers assembled for the load (*P*) and displacement (*h*) of the indenter are of load and displacement resolutions of 1 nN and 0.04 nm, respectively. The crystals were face-indexed through single crystal X-ray diffraction using an Oxford Diffraction Rigaku XtaLAB mini™ diffractometer with Mo-Kα radiation (λ = 0.70173 Å) at 300 K. The experiments are conducted normal to the (00-2), (100) and (110)-oriented faces of the untwined single-crystals with a Berkovich diamond indenter in a quasi-static mode (the tip radius is about 100 nm). The loading and unloading rates of 0.5 mN·s^−1^ and a hold time of 10 s were used. The indentation impressions were captured immediately after unloading which can therefore avoid time-dependent elastic recovery of the residual impressions. The modulus (*E*) and hardness (*H*) values were extracted from each indent using the standard Oliver-Pharr Method and the average values about 15 indents were used in Table 3.

## 4. Conclusions

In summary, we have studied the thermal expansion behavior of framework **1** via variable temperature single-crystal X-ray diffraction. Systematic structural analyses reveal that the large NTE of −26(2) MK^−1^ approximately along its *c*-axis can be explained using the prototypical ‘hinge-strut’-like structural motif. Moreover, the elastic and hardness properties of framework **1** have been explored via nanoindentation experiments and the obtained results reveal its higher mechanical strength compared with other formate frameworks. Furthermore, the mechanical anisotropy of framework **1** can also be understood by referring to the underlying framework structure.

## Figures and Tables

**Table 1:** The coefficients of negative thermal expansion (α, in MK^−1^) for known MFFs.

A	M	α (MK^−1^)	T/K	Reference
CH_3_NH_3_^+^	Mg^2+^	α*_c_* = −20.0(5)	100~300	[14]
	Mn^2+^	α*_c_* = −49(2)		
	Fe^2+^	α*_c_* = −25.1(1.2)		
	Co^2+^	α*_c_* = −28.8(6)		
	Zn^2+^	α*_c_* = −34.6(1.0)		
	Cd^2+^	α*_c_* = −61(7)		
C(NH_2_)_3_^+^	Mn^2+^	α*_c_* = −10.6(3)		
	Fe^2+^	α*_c_* = −1.5(2)		
	Co^2+^	α*_c_* = −6.7(2)		
	Zn^2+^	α*_c_* = −5.3(6)		
	Cd^2+^	α*_a_* = −16.8(9), α*_b_* = −16.8(9)		
[(CH_3_)_2_NH_3_]^+^	Cu^2+^	α*_a_* = −14.3(1.0)		
NH_2_NH_3_^+^	Mg^2+^	α*_a_* = −11, α*_b_* = −69~−89	290~400	[19]
	Mn^2+^	α*_a_* = −96		
	Co^2+^	α*_a_* = −20, α*_b_* = −81~−100	290~405	
	Zn^2+^	α*_a_* = −108	290~375	
	Er^3+^	α*_b_* = −7.1(3)	120~300	[18]
[NH_3_(CH_2_)_4_NH_3_]^2+^	Mg^2+^	α*_b_* = −648	390~410	[17]

**Table 2:** Hydrogen bond (shown in Figure S4) lengths (Å) and bond angles (°) of framework **1** at 120 and 240 K.

D-H⋯A	Lengths (Å) @120 K	@240 K	Angle (deg) @120 K	@240 K
O5 ^v^-H3⋯O1 ^i^	2.769(2)	2.770(2)	169.3(3)	165.0(3)
O5 ^v^-H4⋯O3 ^iii^	2.726(3)	2.746(3)	156.6(4)	150.4(4)
O6 ^vi^-H5⋯O2 ^ii^	2.762(2)	2.762(2)	175.1(4)	170.1(3)
O6 ^vi^-H6⋯O4 ^iv^	2.721(3)	2.727(3)	174.1(4)	162.7(3)

Symmetry code: (i) 2 − *x*, −*y*, 1 − *z*; (ii) 3 − *x*, 1 − *y*, 1 − *z*; (iii) *x*, 0.5 − *y*, 0.5 + *z*; (iv) *x*, 0.5 − *y*, −0.5 + *z*; (v) 2 − *x*, −0.5 + *y*, 1.5 − *z*; (vi) 2 − *x*, 0.5 + *y*, 0.5 − *z*.

**Table 3:** The elastic modulus and hardness properties of some known MFFs.

Metal-Formate Frameworks	D*c* (g·cm^−3^)	Oriention	*E* (GPa)	*H* (GPa)	Reference
[NH_4_][Zn(HCOO)_3_]	1.920	(002)	34.4(9)	-	[7]
		(010)	18.2(2)	-	
[NH_3_NH_2_][Zn(HCOO)_3_]	2.000	(001)	26.5	1.36	[8]
		(110)	24.5	1.24	
[(CH_2_)_3_NH_2_][Mn(HCOO)_3_]	1.735	(010)	12.6(3)	0.66(3)	[22]
		(101)	11.7(3)	0.59(3)	
		(10-1)	11.5(4)	0.58(3)	
[C(NH_2_)_3_][Mn(HCOO)_3_]	1.798	(010)	28.6(4)	1.25(4)	
		(101)	24.5(5)	1.18(4)	
		(10-1)	23.5(6)	1.11(5)	
[NH_2_CHNH_2_][Er(HCOO)_4_]	2.530	(021)	30.2(5)	1.83(5)	[18]
		(02-1)	29.8(8)	1.80(6)	
Framework 1	2.215	(00-2)	35.5(6)	2.04(8)	
		(100)	35.0(9)	1.83(8)	
		(110)	27.1(5)	0.47(3)	

## Supplementary Materials

The following are available online at www.mdpi.com/1996-1944/10/2/151/s1.
