# Supplementary material for: Uniaxial Negative Thermal Expansion and Mechanical Properties of a Zinc-Formate Framework
Source: Materials (Basel). 2017 Feb 10;10(2):151. doi: 10.3390/ma10020151 (PMC5459151; doi:10.3390/ma10020151)
Supplement: Supplementary file 1 [file materials-10-00151-s001.pdf]

Supplementary Materials: Negative Thermal Expansion and Mechanical Properties of a Zinc-Formate Framework

Hongqiang Gao, Wenjuan Wei, Yizhang Li, Rong Wu, Guoqiang Feng and Wei Li

1. Powder X-ray Diffraction

Data was collected in a D8 Advance Bruker AXS h–2 h diffractometer, with a copper radiation source (Cu Ka, k = 1.5406 Å) and a secondary monochromator, operated at 40 kV and 30 mA. The program MERCURY 3.0 was used to obtain the diffraction patterns calculated from SCXRD data. The purity of the bulk was always verified by comparison of the calculated and observed X-ray powder diffraction patterns.

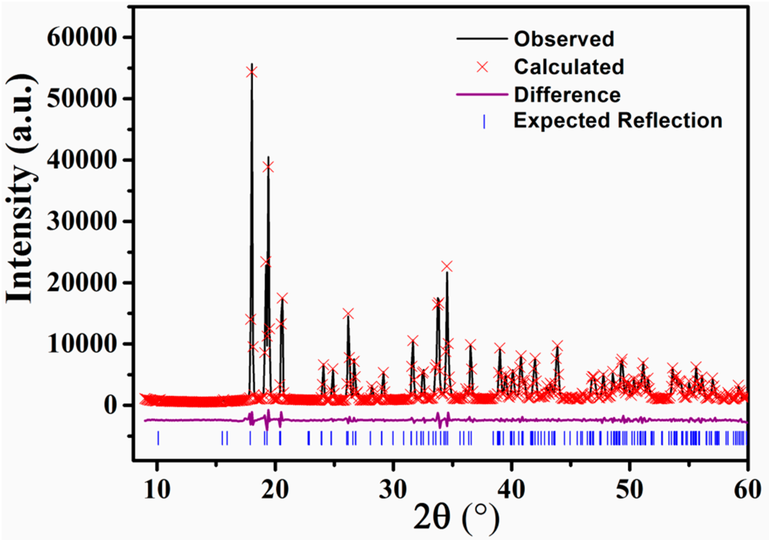

Figure S1. The powder X-ray diffraction (PXRD) spectra of framework **1** refined by the Le-Bail method. The black and red symbols, and purple continuous lines, are the experimental, calculated, and difference profiles, respectively, and the vertical markers indicate the allowed Bragg reflections. R_p = 5.0%, R_{wp} = 7.4%, and GOF = 4.598.

2. The Variations of Volume and Beta Angle

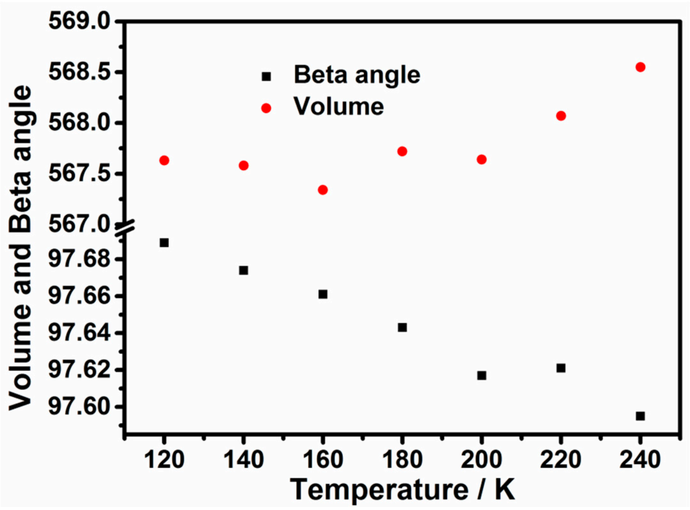

Figure S2. The variations of the volume and beta angle of framework **1** in the 120 K temperature range.

3. Coefficient of Thermal Expansion (α)

The coefficients of thermal expansion (α) of framework **1** are obtained via linear fits using the PASCal program [1]. The expansivity indicatrix of framework **1** is shown in below Figure S3, in which the spatial orientations of the red and blue grid indicate the positive and the negative thermal expansion axes, respectively.

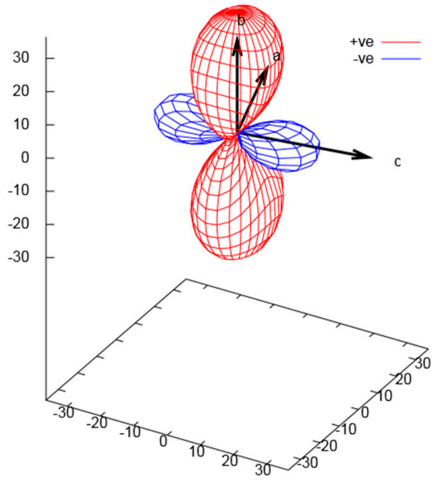

Figure S3. Thermal expansivity indicatrix of framework **1**.

Table S1. The relationships of the principal coefficients of thermal expansion and corresponding principle axes for framework **1**.

		Component of x_i along the Crystallographic Axes			
**Principal Axis, *i***	**α_i (MK^{-1})**	***a***	***b***	***c***	**Approximate Axis**
1	−25.8(2.2)	−0.1841	−0.0000	0.9829	[001]
2	5.3(1.5)	0.9631	−0.0000	0.2693	[100]
3	33.2(0.7)	0.0000	1.0000	−0.0000	[010]

4. The Structure of Framework 1

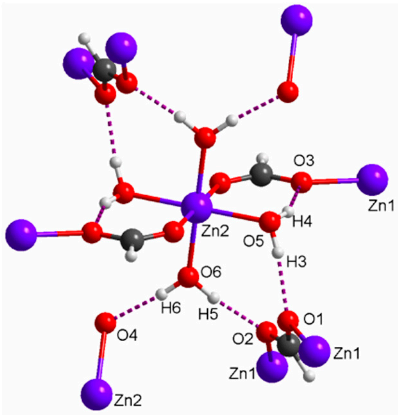

Figure S4. The hydrogen-bonding modes in framework **1**. Symmetry code: (1) $2 - x, -y, 1 - z$; (2) $3 - x, 1 - y, 1 - z$; (3) $x, 0.5 - y, 0.5 + z$; (4) $x, 0.5 - y, -0.5 + z$; (5) $2 - x, -0.5 + y, 1.5 - z$; (6) $2 - x, 0.5 + y, 0.5 - z$.

Reference

1. Cliffe, M.J.; Goodwin, A.L. PASCal: A principal axis strain calculator for thermal expansion and compressibility determination. *J. Appl. Crystallogr.* **2012**, *45*, 1321–1329.
